# Novel neuro-audiological findings and further evidence for *TWNK* involvement in Perrault syndrome

**DOI:** 10.1186/s12967-017-1129-4

**Published:** 2017-02-08

**Authors:** Monika Ołdak, Dominika Oziębło, Agnieszka Pollak, Iwona Stępniak, Michal Lazniewski, Urszula Lechowicz, Krzysztof Kochanek, Mariusz Furmanek, Grażyna Tacikowska, Dariusz Plewczynski, Tomasz Wolak, Rafał Płoski, Henryk Skarżyński

**Affiliations:** 1 Department of Genetics, World Hearing Center, Institute of Physiology and Pathology of Hearing, Mokra 17, Kajetany/Warsaw, 05-830 Nadarzyn, Poland; 2 Laboratory of Functional and Structural Genomics, Centre of New Technologies, University of Warsaw, Warsaw, Poland; 3 Department of Experimental Audiology, Institute of Physiology and Pathology of Hearing, Kajetany/Warsaw, Poland; 4 Bioimaging Research Center, Institute of Physiology and Pathology of Hearing, Kajetany/Warsaw, Poland; 5 Department of Otoneurology, Institute of Physiology and Pathology of Hearing, Kajetany/Warsaw, Poland; 6 Department of Medical Genetics, Medical University of Warsaw, Warsaw, Poland; 7 Oto-Rhino-Laryngology Surgery Clinic, Institute of Physiology and Pathology of Hearing, Kajetany/Warsaw, Poland

**Keywords:** Perrault syndrome, *TWNK*, *C10orf2*, Whole exome sequencing, Auditory neuropathy, Hearing, Vestibulocochlear nerve

## Abstract

**Background:**

Hearing loss and ovarian dysfunction are key features of Perrault syndrome (PRLTS) but the clinical and pathophysiological features of hearing impairment in PRLTS individuals have not been addressed. Mutations in one of five different genes *HSD17B4*, *HARS2*, *LARS2*, *CLPP* or *TWNK* (previous symbol *C10orf2*) cause the autosomal recessive disorder but they are found only in about half of the patients.

**Methods:**

We report on two siblings with a clinical picture resembling a severe, neurological type of PRLTS. For an exhaustive characterisation of the phenotype neuroimaging with volumetric measurements and objective measures of cochlear hair cell and auditory nerve function (otoacustic emissions and auditory brainstem responses) were used. Whole exome sequencing was applied to identify the genetic cause of the disorder. Co-segregation of the detected mutations with the phenotype was confirmed by Sanger sequencing. In silico analysis including 3D protein structure modelling was used to predict the deleterious effects of the detected variants on protein function.

**Results:**

We found two rare biallelic mutations in *TWNK*, encoding Twinkle, an essential mitochondrial helicase. Mutation c.1196A>G (p.Asn399Ser) recurred for the first time in a patient with PRLTS and the second mutation c.1802G>A (p.Arg601Gln) was novel for the disorder. In both patients neuroimaging studies showed diminished cervical enlargement of the spinal cord and for the first time in PRLTS partial atrophy of the vestibulocochlear nerves and decreased grey and increased white matter volumes of the cerebellum. Morphological changes in the auditory nerves, their desynchronized activity and partial cochlear dysfunction underlay the complex mechanism of hearing impairment in the patients.

**Conclusions:**

Our study unveils novel features on the phenotypic landscape of PRLTS and provides further evidence that the newly identified for PRLTS *TWNK* gene is involved in its pathogenesis.

**Electronic supplementary material:**

The online version of this article (doi:10.1186/s12967-017-1129-4) contains supplementary material, which is available to authorized users.

## Background

Perrault syndrome (PRLTS) is suspected in a female with bilateral sensorineural hearing loss (SNHL), ovarian dysfunction that may occur in a mild (primary ovarian insufficiency) or severe (ovarian dysgenesis) form and a normal 46,XX karyotype [1]. In males PRLTS is manifested by SNHL and their fertility is generally considered normal but this matter should be regarded with caution due to scarcity of males with PRLTS and data from the animal studies [2, 3]. SNHL is usually the initial expression of PRLTS. In some patients a spectrum of neurologic symptoms develops over time indicating a progressive, age-related neurodegenerative process.

Common neurological abnormalities are motor and sensory neuropathy, muscle weakness and atrophy, hypo- or areflexia, cerebellar ataxia, limited eye movements, nystagmus, dyspraxia, as well as intellectual deficit, developmental delay and seizures [4–7]. In brain magnetic resonance imaging (MRI), nonspecific white matter changes suggestive for cerebral leucodystrophy and cerebellar atrophy are described [5, 6, 8]. Depending on the occurrence of neurologic features, a classification of PRLTS into type I, static and without neurologic involvement and type II with a progressive neurological disease has been proposed [7].

Transmission of PRLTS is autosomal recessive and over the last years five genes causative for PRLTS have been identified. Mutations in *HSD17B4* (OMIM *601860; PRLTS1), encoding a peroxisomal enzyme, were found in four PRLTS families [9–12]. Mutations in *HARS2* (OMIM *600783; PRLTS2) were detected in three families [4, 13], mutations in *LARS2* (OMIM *604544; PRLTS4) in six families [10, 13–15], mutations in *CLPP* (OMIM *601119; PRLTS3) in seven families [5, 10, 13, 16, 17], and mutations in *TWNK* (OMIM *606075; PRLTS5) only in four families with PRLTS [6, 10, 13]. All of the four latter PRLTS genes code for mitochondrial proteins. Molecular etiology for PRLTS remains unknown in as many as 55% of the patients, which emphasizes a genetic heterogeneity of the syndrome and shows that novel disease-causing genes still await discovery [10, 13].

Twinkle mtDNA helicase (*TWNK*), located in the long arm of chromosome 10 (10q24.31), encodes Twinkle, which localizes to mitochondrial nucleoids and forms a hexameric or heptameric ring structures. Twinkle is composed of a primase and helicase domain connected via a linker region, involved in stabilization of the oligomeric complexes of the protein. With the primase activity the protein initiates DNA replication and with the helicase activity unwinds DNA for replication. Twinkle is essential for the replication process of mitochondrial DNA (mtDNA) and lifetime maintenance of human mtDNA integrity [18, 19].

In this study, whole-exome sequencing (WES) was successfully applied to identify the molecular basis of PRLTS in the proband and her sister from a non-consanguineous Polish family. The cardinal manifestations of PRLTS (SNHL and ovarian dysgenesis) in the patients were preceded and dominated by severe and progressive involvement of the nervous system, making the diagnosis not straightforward. Here, we provide a thorough neurological and audiological assessment of both patients that unveil novel features on the phenotypic landscape of PRLTS.

## Patients and methods

### Study subjects

Two affected sisters from a Polish non-consanguineous family and their unaffected mother were available for the study. The proband was born at term by Cesarean section after an uneventful pregnancy with 3600 g of body weight and an Apgar score of 1/2/3, indicating severe birth depression. Her psychomotor development was normal until the third year of age, when the motor performance begun to deteriorate progressively and the electromyography (EMG) examination was abnormal (she is unable to run since the age of 15 years). Speech development was age appropriate. At 5 years of age SNHL was diagnosed and pathologic auditory brainstem responses (ABRs) were observed. She has been fitted with hearing aids at the age of 13 but they were of limited benefit. Testing for primary amenorrhea and delayed pubertal development at the age of 15 revealed hypergonadotropic hypogonadism, streak gonads, rudimentary uterus and a normal female karyotype 46,XX. Hormone replacement therapy was introduced. At the age of 16 chronic thyroiditis with elevated levels of anti-thyroid peroxidase antibodies was diagnosed. Nerve conduction studies at the age of 21 showed axonal sensorimotor polyneuropathy. Ophthalmological examination was unremarkable except for impaired eye movements (Table 1).

**Table 1:** Clinical features and laboratory findings in the affected family members

Clinical features	Proband	Sister
Sex	F	F
Age at disease onset, years	3	11
Age at examination, years	27	19
Disease duration	24	8
Sensorineural hearing loss	+(5*)	+(12*)
Ovarian dysfunction	+(15*)	+(12*)
Intellectual disability	–	–
Dementia	–	–
Epilepsy	–	–
Cerebellar syndrome	+(3*)	+(11*)
SDFS	3	2
Impaired eyes movement	+	+
Gaze-evoked horizontal nystagmus	+	+
Gaze-evoked vertical nystagmus	–	+
Dysarthria	+	+
Ataxia	+	+
Positive Romberg’s test	+	+
Flaccid paresis	+(?*)	+(?*)
Muscle weakness	+UL < LL	+UL < LL
Muscle atrophy	+UL < LL	+UL < LL
Tendon reflexes UL	Diminished	Diminished
Tendon reflexes LL	Absent	Absent
Gait	Steppage	Steppage
High-arched palate	+	+
Pes cavus and clawed toes	+	+
Other features—Hashimoto disease	+	+

Laboratory findings		
Lactate elevation	n.a.	+
CK elevation	n.a.	+
FSH and LH elevation	+	+
EMG	Axonal, sensorimotor polyneuropathy	Axonal, sensorimotor polyneuropathy
Abnormal neuroimaging	+	+

Numbers in brackets (*) refer to age at diagnosis, years

*UL* upper limbs, *LL* lower limbs, *n.a.* no data available

The proband’s sister was born at term with 2850 g of body weight and an Apgar score of 5/6/7. Horizontal nystagmus and imbalance began at the age of 11, walking was gradually deteriorating and sensorimotor polyneuropathy was identified. At the age of 12 SNHL and ovarian dysgenesis with normal female karyotype were diagnosed. From the age of 16 years she has a Hashimoto’s disease. Biochemical studies showed mildly elevated levels of serum lactate and creatine kinase. Metabolic disease screening for organic acids with gas chromatography-mass spectrometry (GS/MS) in urine, amino acids and acylcarnitines with liquid chromatography-tandem mass spectrometry (LC–MS/MS) in dried blood spot and congenital disorders of glycosylation gave normal results. Wilson and Refsum diseases were excluded based on normal blood concentrations of ceruloplasmin and cooper and phytanic acid, respectively.

### Neurological and audiological evaluation

Written informed consent was obtained from each participant. The study was approved by the ethics committee at the Institute of Physiology and Pathology of Hearing and performed according to the Declaration of Helsinki. The patients underwent thorough clinical evaluation. Functional impairment was assessed according to spinocerebellar degeneration functional score (SDFS) [20]. In brain and cervical spine MRI (3T Siemens Magnetom Trio, 12-channel Head Matrix Coil) T1-weighted, T2-weighted and diffusion-weighted images were acquired. In addition, high-resolution 3D structural T1-weighted volumes were acquired using an MPRAGE sequence with 208 sagittal slices and an isotropic resolution 0.9 × 0.9 × 0.9 mm. Sequence parameters were: TR = 1900 ms, TE = 2.21 ms, TI = 900 ms, FA = 9, FOV = 26 × 28.8 cm, matrix = 320 × 290, Pixel bandwidth = 200 Hz/pix, iPAT = 2, TA = 5 min. Brain structures were segmented by Freesurfer version 5.3 (http://surfer.nmr.mgh.harvard.edu/). The diameter of the vestibulocochlear nerve was evaluated directly and compared with the neighboring facial nerve used as an internal reference. Assessment of the cochlear and vestibular components was based on their visual comparison and with reference to the facial nerve. For comparisons of the cerebrum and cerebellum volumes and their white and gray matters, age- and sex-matched controls [21–23] from the Internet Brain Volume Database funded by The Human Brain Project (http://ibvd.virtualbrain.org/) were used. A difference above 2.3 of the standard deviation was considered as statistically significant.

Assessment of auditory function consisted of pure-tone and speech audiometry, impedance audiometry, otoacoustic emissions (OAE) and ABRs. Hearing thresholds for air and bone conduction were determined at frequencies 125–8000 and 250–4000 Hz, respectively, using the AC40 clinical audiometer (Interacoustics, Middelfart, Denmark) and the 10/5 dB descending-ascending threshold estimation procedure [24]. Speech comprehension was tested using monosyllabic Polish words and the AC40 audiometer (Interacoustics) [25]. Acoustic impedance measurements (tympanograms and stapedius reflex) were performed with the Zodiac 901 instrument (Madsen Electronics, Copenhagen, Denmark). Stapedius reflexes were analyzed for the frequencies 500, 1000, 2000 and 4000 Hz in the ipsi and contralateral modes [26]. OAE were evoked by standard-click stimuli and 500 Hz tone bursts by using the ILO-292 system (Otodynamics Ltd, Hatfield, United Kingdom) [27–29]. ABRs were recorded using the Integrity V500 system (Vivosonic Inc., Toronto, Canada). The stimuli were 0.1 ms clicks with alternating polarity presented with 90 dB normal hearing level (nHL) intensity at a repetition rate of 11/s. The amplifier bandwidth was 30–1500 Hz and analysis time 12 ms. The number of sweeps required for an averaged response was 1024 [30].

For evaluation of the vestibular endorgan function Fitzgerald and Hallpike bithermal caloric test with video eye movement recordings (Visual Eyes Micromedical Technologies, Chatham, USA) were used. Sinusoidal harmonic acceleration testing at frequencies 0.01–0.32 Hz was conducted with a Rotational Vestibular Chair System 2000 (Micromedical Technologies, Chatham, USA). Otolith function was measured using air-conducted sound stimulation cervical and ocular vestibular evoked myogenic potentials (cVEMP, oVEMP) at 500 Hz, 95 dBnHL (EclipsVemp, Interacoustics, Assens, Denmark).

### Whole-exome and Sanger sequencing

DNA was isolated from blood sample by a standard procedure. WES was performed using SureSelect Target Enrichment (Agilent Technologies, Palo Alto, CA, USA) according to the manufacturer’s protocol. The sample was run on 16% of a lane on HiSeq 1500 using 2 × 100 bp paired-end reads. All bioinformatics analysis was done as described previously [31]. After primarily CASAVA processing, all reads were aligned to the hg19 reference genome with the Burrows-Wheeler Alignment Tool and analyzed with Genome Analysis Toolkit [32]. Indel realignment, base quality score recalibration, duplicates elimination as well as SNP/INDEL calling were performed [33]. The retrieved variants were annotated with ANNOVAR and converted to MS Access format for subsequent manual analyses. Total exon coverage by 20 reads or more was 89% and by 10 reads or more 95.9%. Alignments were inspected with Integrative Genomics Viewer [34] and analyzed with a pipeline combining protein coding changes, splice site prediction, prevalence in populations, evolutionary conservation and scores from PolyPhen-2 [35], SIFT [36] and MutationTaster2 [37] prediction algorithms of non-synonymous single-nucleotide variants. Sanger sequencing with 3500xL Genetic Analyzer (Applied Biosystems, Foster City, CA, USA) and BigDye Terminator cycle sequencing kit v. 3.1 (Applied Biosystems) were used to confirm the presence of variants identified by WES.

### In silico protein analysis

Homologs of the human Twinkle protein were identified with a PSI-Blast [38] search (E-value threshold of 0.005) performed against the NCBI non-redundant protein sequence database. The collected 15,000 sequences were initially clustered at 60% sequence identity with cd-hit [39] and any sequences shorter than 300, longer than 1000 amino acids or described as “hypothetical protein” were removed. The multiple sequence alignment of the twinkle family was derived using MAFFT program [40].

For preparation of the Twinkle homology model, the crystal structure of the gp4 protein from bacteriophage T7 (Protein Data Bank code1e0j) was selected as a template after analyzing the GeneSilico Metaserver results [41]. The sequence-to-structure alignment between the Twinkle protein and the template (Additional file 1: Figure S1) was built using the consensus alignment approach and 3D assessment [42] based on the results of FFAS [43], HHSearch [44] and the alignment proposed by Fernandez et al. [19]. Multiple sequence alignment of the family was also taken into consideration. The 3D model of the protein was built with MODELLER [45]. A model quality assessment was carried out using ProSA-web server [46]. Secondary structure elements were predicted with PSI-PRED [47]. Structure visualization was carried out with PyMOL (http://www.pymol.org).

## Results

### Involvement of the nervous system

The proband and her sister came to our observation at the age of 27 and 19, respectively. They both had progressive neurologic symptoms, including postlingual progressive SNHL, cerebellar syndrome and flaccid paresis (Table 1) with muscle atrophy being more prominent in the proband.

**Fig. 1:**
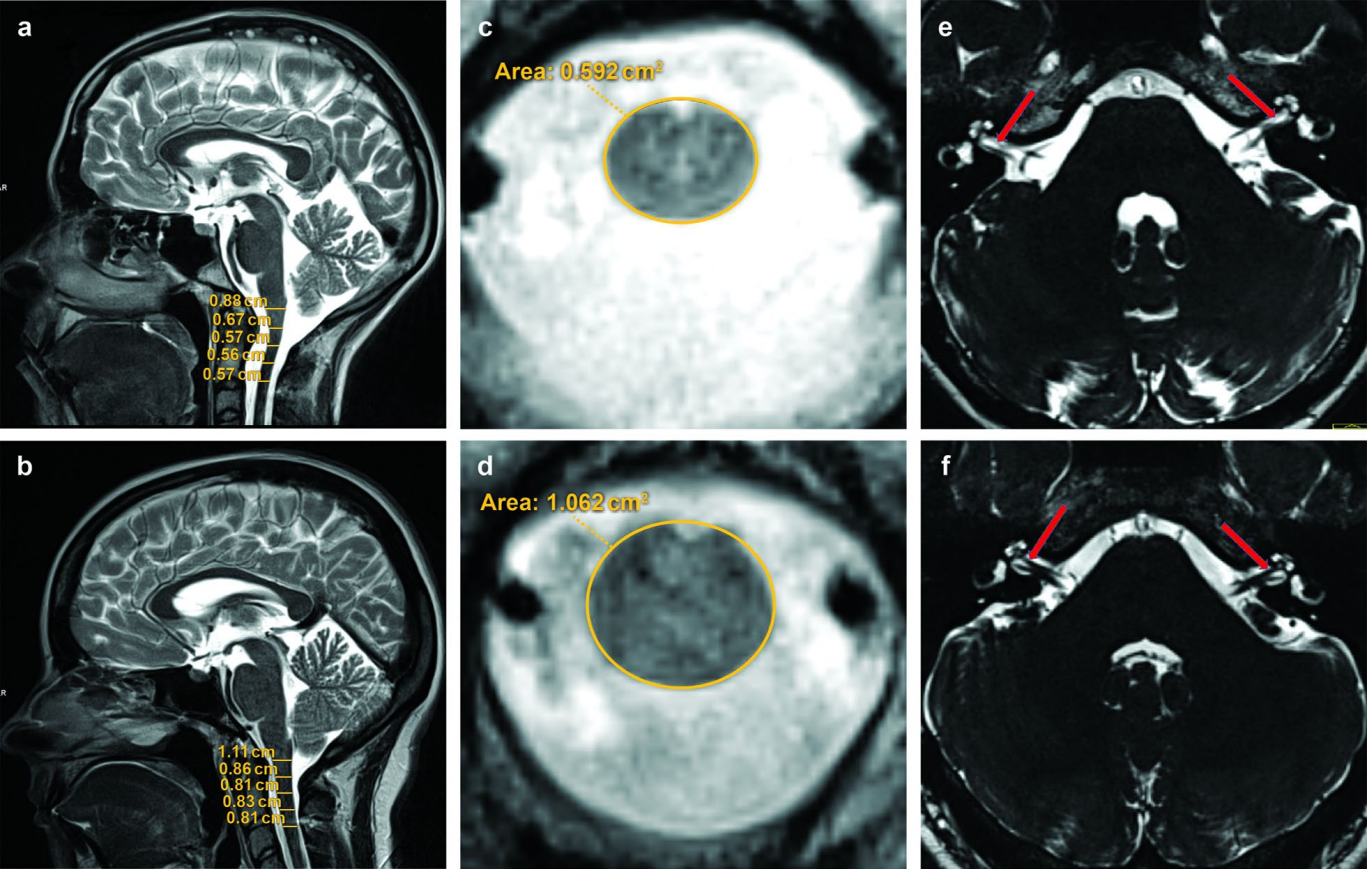
Brain and spine MRI in the proband. The *left column* shows sagittal T2 weighted images of the head and cervical spine (**a** proband, **b** control) with the measurements of spinal cord thickness at different levels. The *middle column* shows cross-section of the spinal cord (**c** proband, **d** control). The *right column* shows transverse heavy T2 weighted images of sub-millimeter slice thickness displaying the cochlear nerve, pointed with a* red arrow* (**e** proband, **f** control)

Brain MRI in both sisters revealed considerable bilateral thinning of the vestibulocochlear nerve and its cochlear and vestibular components, which was defined as partial atrophy. It was more pronounced in the cochlear than in the vestibular nerves. Morphological signs of a subtle cerebellar atrophy (widening of the sulci more pronounced in the vermis than in hemispheres) were found in the proband but not in the sister. In cervical spine MRI, a diminished cervical enlargement was observed in both patients (Fig. 1). In the proband volumetric measurements of the brain showed significantly increased cerebellum white and decreased cerebellum gray matter. The results of other volumetric measurements were unchanged (Table 2).

**Table 2:** Volumes of cerebrum, cerebellum and the respective gray and white matters in the proband and her sister

Brain part	Proband (cm^3^)	Control groups (cm^3^)^a^	Proband’s sister (cm^3^)	Control group^b^ (cm^3^)
Cerebrum volume	1030	1181.3 ± 83.1	1187.1	1172 ± 108.1
		1148.5 ± 77.2		
Cerebrum gray matter volume	571	672.3 ± 33.3	674.2	579 ± 50.6
		663.2 ± 51		
Cerebrum white matter volume	406	384.6 ± 40.5	464.2	435 ± 50.6
		425.3 ± 30		
Cerebellum volume	131.1	153.8 ± 10.8	145.2	142 ± 17.5
		134.7 ± 6.8		
Cerebellar gray matter volume	98.4	128.1 ± 9.2	111.9	n.a.
		112.3 ± 5.9		
Cerebellar white matter volume	32.7	26 ± 2.2	33.7	n.a.
		22.4 ± 2		

*n.a.* no data available

^a^Control groups from two different studies: first values [21], second values [22]

^b^Control group from [23]; underlined are statistically significant differences from both control groups

### Functional alterations in vestibulocochlear pathway

**Fig. 2:**
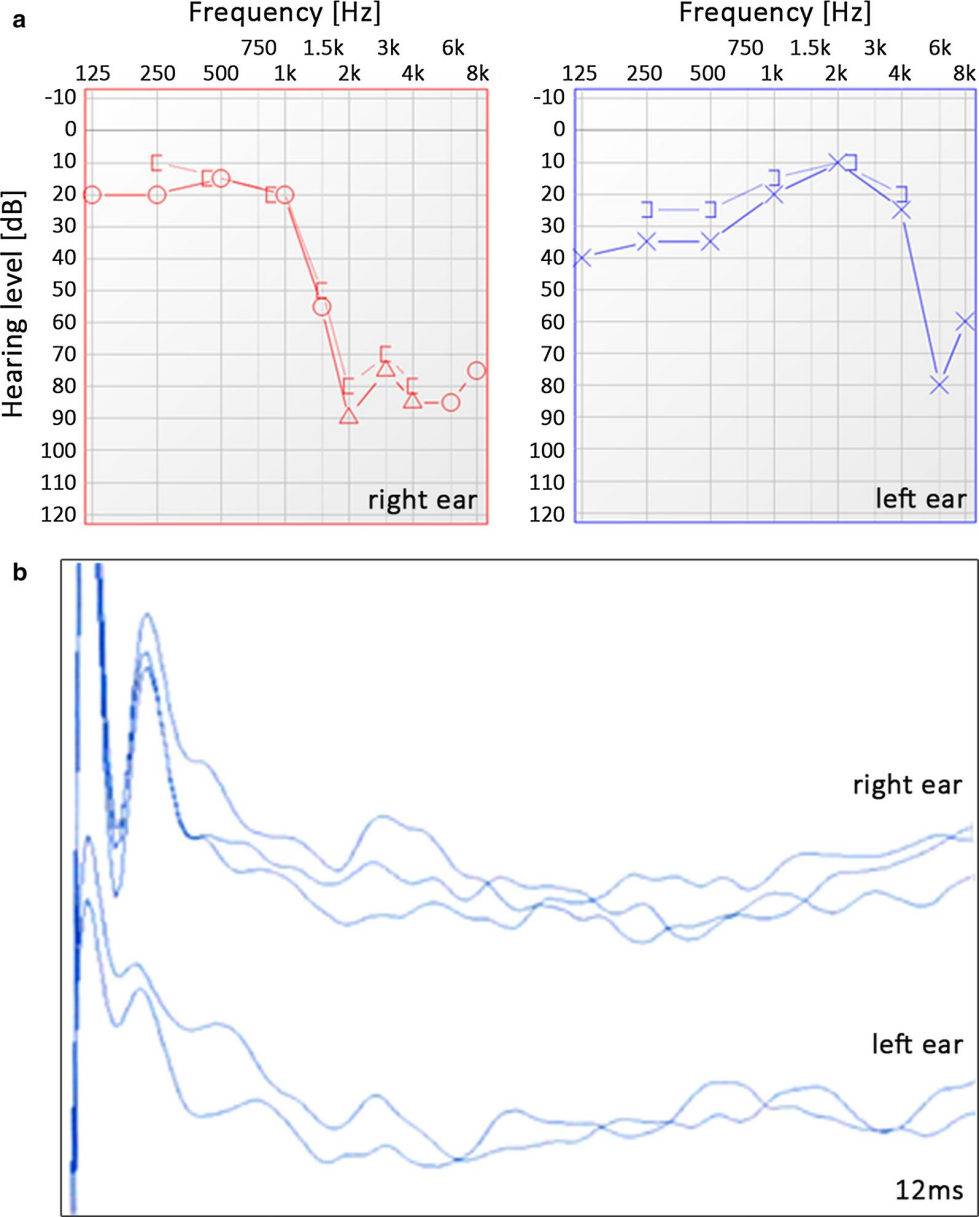
Pure tone audiometry (**a**) and ABRs (**b**) of the proband. **a** “O” and “X” symbols denote air conduction thresholds in the *right* and *left ear*, respectively and “Δ” denotes masked air conduction at high frequencies in the *right ear*; “[” or “]” denote masked bone conduction. **b** Shown are ABR recordings after click stimulus at an acoustic level of 90 dB normal hearing level (nHL) and presentation rate of 11/s

In the proband and her sister, a different degree of sensorineural hearing loss mainly affecting high frequencies has been diagnosed in pure-tone audiometry (Fig. 2a). In contrast to pure-tone thresholds, speech discrimination was unproportionally poor and the test has not been continued. In both patients, the tympanograms revealed normal middle ear function. Ipsi- and contralateral acoustic reflexes were absent for all tested frequencies. The analysis of OAE recordings from both patients showed the presence of otoacoustic emission signals in the frequency range up to 2 kHz in the right ear and up to 4 kHz in the left ear. OAE in both patients were largely consistent with the results of pure-tone audiometry, demonstrating a partially impaired function of the outer hair cells. In ABR recordings, no responses at the maximum level of 90 dB nHL were obtained biaurally in the proband (Fig. 2b) and her sister. Comprehensive audiological evaluation revealed auditory neuropathy that was accompanied by a certain degree of cochlear dysfunction.

In both patients bi-thermal caloric irrigation results were within the normal limit. Rotational testing showed abnormally reduced gain and increased phase at 0.01, 0.02 and 0.04 Hz and was unaffected at the remaining frequencies. In the proband cVEMP and oVEMP were not recorded on either side. In her sister only oVEMP on the left side was registered with a prolonged latency of N1 and P1 peaks (data not shown). The results are suggestive of a neuropathy of both the superior and the inferior vestibular nerves in addition to the involvement of the auditory branch of the vestibulocochlear nerve.

### Identification of compound heterozygous *TWNK* mutation

**Fig. 3:**
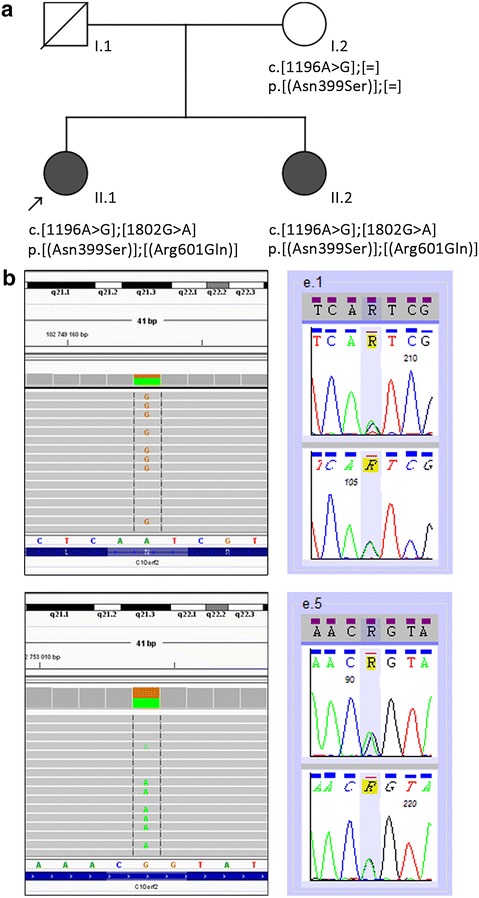
Identification of *TWNK* mutations in the analyzed families. **a** Pedigree of the investigated family. The proband is marked with an *arrow*. *Black symbols* indicate individuals affected with PRLTS5 and *open symbols* indicate unaffected individuals; *diagonal line* denotes the deceased father. The *TWNK* genotypes identified in the family members are reported at the cDNA and protein levels according to the HGVS-nomenclature (http://varnomen.hgvs.org/; accessed 07/2016). **b** WES in the proband revealed an A>G transition (*upper left panel*) and G>A transition (*lower left panel*), corresponding to p.Asn399Ser (AAT>AGT) and p.Arg601Gln (CGG>CAG), respectively, in the *TWNK* gene. Direct Sanger sequencing of *TWNK* confirmed the presence of the two mutations (*right panel*). For each mutation sequencing of the forward (*top*) and the reverse (*bottom*) strand is shown

DNA sample of the proband was analyzed by whole exome sequencing. After exclusion of variants found with a prevalence of 1% or more in the databases of the Exome Aggregation Consortium (ExAC, http://exac.broadinstitute.org/), 1000 Genomes Project (http://www.1000genomes.org) and the NHLBI GO Exome Sequencing Project (ESP, http://evs.gs.washington.edu/EVS/; all accessed 05/2016) and in a set of 816 exomes of Polish patients (ZGM, R. Płoski, unpublished results) in the first line we searched for variants reported in the Human Gene Mutation Database (www.hgmd.cf.ac.uk/ac/index.php) and variants predicted to be pathogenic by bioinformatic tools. We found a rare *TWNK* heterozygous missense variant NM_021830.4:c.1196A>G (rs863223921), causing the missense change NP_068602.2:p.Asn399Ser (Fig. 3a, b) that has been identified for the first time in 2016 in a Norwegian female with PRLTS [10] (Table 3). The variant is predicted to be damaging by PolyPhen-2 (score 0.993), SIFT (score 0.02) and MutationTaster2 (score 0.998).

**Table 3:** Comparison of demographic and molecular findings in PRLTS patients with *TWNK* mutation

Family # and origin	Consanguinity	PRLTS	Reference sequence number	Mutation cDNA level	Mutation protein level	Karyotype	Reference
1. Japanese	N	Two sisters	rs556445621	c.1172G>A	p.Arg391His	46,XX	[6]
			rs672601360	c.1754A>G	p.Asn585Ser		
2. American with European ancestry	N	Two sisters	rs672601361	c.1321T>G	p.Trp441Gly	46,XX	[6]
			rs369588002	c.1519G>A	p.Val507Ile		
3. Norwegian	N	One female	rs770917763	c.968G>A	p.Arg323Gln	46,XX	[10]
			rs863223921	c.1196A>G	p.Asn399Ser		
4. Moroccan	Y	Two sisters and brother	rs764669712	c.793C>T	p.Arg265Cys	n.a.	[13]
			rs764669712	c.793C>T	p.Arg265Cys		
5. Polish	N	Two sisters	rs863223921	c.1196A>G	p.Asn399Ser	46,XX	Present study
			rs141315771	c.1802G>A	p.Arg601Gln		

*N* no, *Y* yes, *n.a.* no data available

The second *TWNK* variant was a very rare heterozygous missense change NM_021830.4:c.1802G>A (rs141315771), causing the amino acid substitution NP_068602.2:p.Arg601Gln (Fig. 3a, b). The p.Arg601Gln variant was reported only in the ExAC and ESP databases (accessed 07/2016) with an allele frequency of 3.29e−5 (4/121412 alleles) and 1.54e-4 (2/13006 alleles), respectively but heretofore it has not been identified in a homozygous state or associated with any disease. The G>A transition is predicted to be damaging by PolyPhen-2 (score 0.895), SIFT (score 0.02) and MutationTaster2 (score 0.997).

Presence of the two heterozygous *TWNK* mutations was confirmed in the proband’s sister. The mother was a carrier of the p.Asn399Ser mutation, showing that the two *TWNK* mutations are biallelic and the patients are compound heterozygous for the mutations (Fig. 3a, b). DNA sample from the deceased father was not available for the study.

### Modelling of p.Asn399Ser and p.Arg601Gln functional roles

**Fig. 4:**
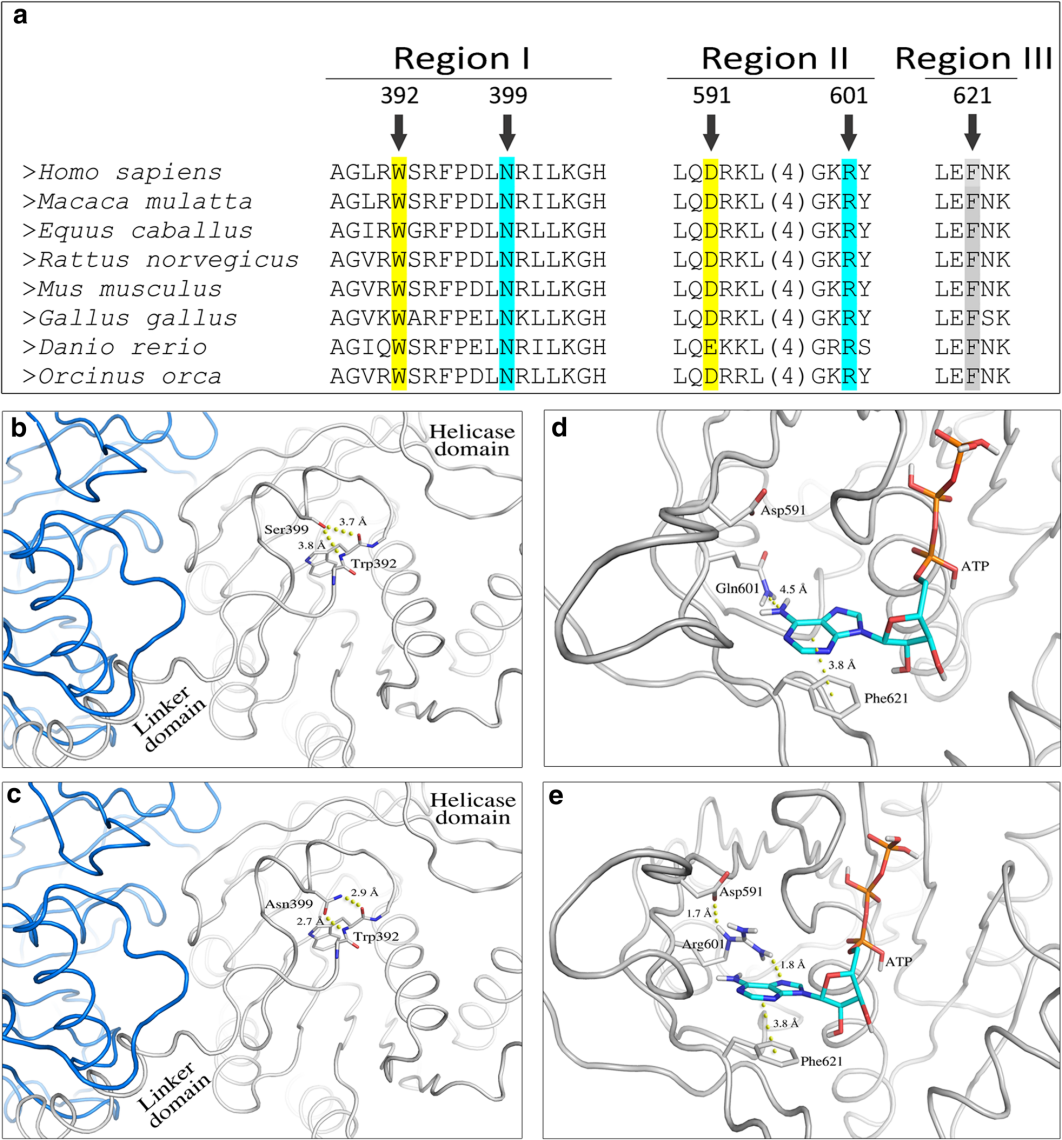
Multiple protein sequence alignment and 3D structure of the human Twinkle protein in regions encompassing p.Asn399Ser and p.Arg601Gln mutations. **a** Multiple protein sequence alignment of selected sequences. Two regions of the twinkle protein are shown: (i) the region joining the linker and the helicase domains (*Region I*) and (ii) the region involved in stabilizing the adenine ring of ATP (*Regions II* and *III*). The numbers above the alignment correspond to the amino acid position in the human protein sequence. (**b–c**) Two interacting monomers of the Twinkle protein are colored *white* and *blue*. Changes in the conformation of a region next to the linker domain in p.Asn399Ser mutant (**b**) and in wild type (**c**) proteins are shown. (**d–e**) Changes in hydrogen bonds network resulting in weakened ATP binding in the p.Arg601Gln mutant (**d**) as compared to wild type (**e**) protein are depicted

Multiple sequence alignment demonstrated that Asn399 and Arg601 are conserved amino acid residues among vertebrates (Fig. 4a). In the crystal structure of the bacteriophage T7 gp4 protein (human Twinkle homolog), Asn289 (human Asn399) forms two hydrogen bonds with the backbone of Phe296 (human Trp392). A similar scenario is observed for the human protein. The side chain of Asn399, which is located on the short helix, forms two hydrogen bonds with the backbone atoms of Trp392. Both amino acids are located in a region between the linker and helicase domains. The p.Asn399Ser mutation disrupts these interactions as Ser399 is located too far away to form hydrogen bonds with the main chain of Trp392 (Fig. 4b, c). As a result, the entire region may adopt a different conformation than in the wild type protein. This may impair the correct orientation of the linker region and hinder the hexamer/heptamer formation [19].

In the T7 gp4 protein the adenine ring of the ATP analog is stacked between Arg504 (human Asp591) and Tyr535 (human Phe621) [48]. Val514 (human Arg601) is positioned next to the adenine ring and placed on the same plane. In the human Twinkle protein the key Arg504 is replaced by an aspartic acid, which is unable to stabilize the adenine ring. Simultaneously, an arginine (Arg601) is introduced at the position occupied by a short-chained amino acid (Val514) in the T7 phage. According to our 3D model, human Arg601 could adopt a conformation, which allows the formation of a hydrogen bond with the ATP N7 atom. This conformation can be stabilized by hydrogen bond interactions with Asp591. In contrast to the T7 phage Arg504, human Arg601 seems not to form a cation-π interaction with the adenine ring. However, we cannot exclude the possibility that the loop, on which both Arg601 and Asp591 are located, assumes a different conformation than in our model. This may allow Arg601 to adopt a conformation where the adenine ring is stabilized via stacking rather than hydrogen bonding. The interaction between ATP and Phe621 remains unaffected. Substitution of Arg601 to Gln most likely weakens the binding of ATP as Gln is unable to form a hydrogen bond with the adenine N7 atom (Fig. 4d, e).

## Discussion

Herein, we report the identification of a distinctive phenotype of PRLTS5 (OMIM #616138), in which the progressive neurologic features, dominating in the phenotype, preceded the diagnosis of SNHL and ovarian dysfunction. Comprehensive analysis of the patients’ phenotype and family history enabled us to establish a clinical suspicion of PRLTS. After applying WES the underlying cause of the disorder has been explained by the detection of compound heterozygous mutations in the *TWNK*. It is the most recently discovered gene involved in the pathogenesis of PRLTS that heretofore has been reported only in four PRLTS families worldwide.

Pathogenic role of heterozygous *TWNK* mutations have been first discovered in families with autosomal dominant progressive external ophthalmoplegia (PEOA3; OMIM #609286) [18]. Recessive *TWNK* mutations are causative for mitochondrial DNA depletion syndrome 7 (MTDPS7; OMIM #271245) also known as infantile-onset spinocerebellar ataxia (IOSCA) [49, 50] and were recently identified in patients with PRLTS5 [6, 10] (Table 3). There is a substantial phenotypic overlap across the conditions resulting from *TWNK* mutations, particularly in regard to the neurological features. Hearing loss, ataxia, myopathy, neuropathy and ophthalmoplegia have been reported in patients with each of these diseases. In our patients the diagnosis of PEOA3 could be excluded based on the presence of ovarian dysgenesis that has not been described in patients with PEOA3, an earlier age of disease onset (1–2 vs. 2–8 decade of life) and the identification of two *TWNK* mutations that were not pathogenic in the patients’ parents (heterozygous carriers). In contrast, MTDPS7 begins very early in life, in children below 2 years of age; the course of the disease is severe and includes optic atrophy, intellectual disability and hepatic involvement that were not observed in our patients.

All mutations found in *TWNK* are missense changes and their location does not explain different clinical manifestations. It has been hypothesized that even slight disturbances to the Twinkle protein, as a consequence of *TWNK* mutation, may affect its enzymatic activity, DNA binding ability, interaction with subunits or stability and result in a less-effective enzyme [51, 52]. Different bioinformatics tools predicted a deleterious effect of p.Asn399Ser and p.Arg601Gln on the protein function. In the applied 3D model we showed that p.Asn399Ser may affect the oligomeric Twinkle structure, crucial for the enzyme’s ability to unwind the DNA. A consequence of p.Arg601Gln appears to be impaired binding and hydrolysis of ATP, which is of paramount importance for enzyme functioning.

No formal criteria have been elaborated to facilitate accurate recognition of PRLTS. In the first line, karyotype analysis should be performed to exclude Turner syndrome or other abnormalities of the X chromosome as approximately half of females with Turner syndrome (gonadal dysgenesis) suffer from hearing loss [53]. Next, other causes of sensorineural hearing loss (SNHL) and ovarian dysfunction should be considered. Both conditions are genetically heterogeneous and testing of causative mutations in known genes is appropriate. In patients with neurologic involvement, the phenotype of PRLTS may overlap clinically with a mild form of peroxisomal d-bifunctional protein deficiency (DBP type IV; OMIM #261515). Presence of ovarian dysgenesis is considered the major clinical feature differentiating PRLTS from DBP type IV [9]. A comprehensive analysis of WES data provided us with a wealth of information on the genetic constitution of the proband. Except for two *TWNK* mutations no other mutation has been identified, which alone or in combination with other pathogenic variants could account for the clinical features observed in the proband. After identifying both *TWNK* mutations in the proband’s sister and confirming their biallelic status we could unequivocally establish the molecular genetic basis of the disease in the studied family. Our results provide further evidence that mutations in *TWNK* cause PRLTS5 [6, 10].

Neurological features in PRLTS have been observed in patients with mutations either in *HSD17B4*, *CLPP* or *TWNK* genes, i.e. in three out of five known PRLTS genes [5, 6, 9–12, 16, 17]. However, involvement of the nervous system seems to be a constant finding only in patients with *TWNK* mutations. In contrast to our patients in other individuals with PRLTS5 the neurological problems became noticeable later in life after hearing loss and ovarian dysfunction have been diagnosed [6, 10, 13]. The proband had a subtle atrophy of the cerebellum, a feature previously described in PRLTS patients [9, 10]. Significantly distorted proportion between the cerebellum white and grey matters represents a novel finding in PRLTS patients, which was independently confirmed using two different control groups. Atrophy of cervical medulla, present in both of our patients, was a feature of another PRLTS5 patient, who also shared the *TWNK* p.Asn399Ser mutation [10].

Audiological examination and neuroimaging studies revealed that hearing loss in PRLTS5 patients has a complex background. Analysis of the hearing threshold together with OAE (absent at higher frequencies) pointed to impairment of the cochlear function. While lack of stapedius reflexes and ABRs, together with normal tympanometry and the presence of OAE, were classical features of auditory neuropathy. Absent ABRs suggested that the defect localizes to the synapse between the hair cells and the auditory nerve. As imaging studies revealed partial atrophy of the vestibulocochlear nerve, their vestibular and cochlear components and the patients manifested peripheral neuropathy, we assume that the disease process affects the vestibulocochlear nerve fibers, particularly their distal parts. It should be also taken into account that absent ABRs may represent a progressed state of the disease, which has begun in the proximal part of the vestibulocochlear nerve as it is observed in other hereditary neurological diseases affecting mitochondrial function such as Friedreich’s ataxia or Charcot–Marie–Tooth (excluded in the proband based on genetic tests) [54, 55].

Partial atrophy of the vestibular nerves was less marked than the atrophy of the cochlear nerve and functionally sufficient to provide normal results of the vestibulo-ocular reflex in caloric tests and to some extend of the rotational testing. However, the number of vestibular nerve fibers discharging synchronously appears insufficient to obtain normal results of VEMP responses. It should be also considered that abnormal or absent VEMPs could be a consequence of the neuropathological process affecting the medial vestibulospinal tract (VEMP descending pathway) as diminished cervical enlargement was found in both patients. Asymptomatic vestibular disorders are commonly observed in patients with auditory neuropathy accompanied by peripheral neuropathy [56].

## Conclusions

Our study presents a detailed and systematic assessment of the auditory and nervous systems in patients with PRLTS5, which sheds new insight on the phenotype and disease process of the mitochondrial disorder. We show for the first time in PRLTS patients (1) a complex mechanism of hearing impairment, comprising of cochlear dysfunction and dyssynchronous auditory nerve function, (2) partial atrophy of the vestibulocochlear nerves and their auditory and vestibular parts and (3) aberrant proportion of the cerebellum white and grey matters. Genetic workup provided further evidence on the causative role of *TWNK* mutations. From the seven known *TWNK* mutations underlying PRLTS5 one has recurred in our patients and its detection in the second individual with PRLTS5 confirms the pathogenic potential of the variant. The second *TWNK* mutation has not been reported in the context of any disease and its identification represents a novel genetic association with PRLTS5.

## Additional file

**Additional file 1: Figure S1.** Sequence-to-structure alignment between the human Twinkle protein (Hs) and the template (PDB code 1e0j). The numbers of residues that are not shown are specified in parentheses. Identical residues are highlighted in yellow. Locations of observed (for 1e0j) and predicted (for Hs) secondary structure elements are marked above and below the corresponding sequences. Helices are presented as blue cylinders, while beta strands as orange arrows.

## Abbreviations

PRLTS: Perrault syndrome
SNHL: bilateral sensorineural hearing loss
MRI: magnetic resonance imaging
*TWNK*: twinkle mtDNA helicase
mtDNA: mitochondrial DNA
WES: whole-exome sequencing
EMG: electromyography
ABRs: auditory brainstem responses
GS/MS: gas chromatography-mass spectrometry
LC–MS/MS: liquid chromatography-tandem mass spectrometry
SDFS: spinocerebellar degeneration functional score
OAE: otoacoustic emissions
nHL: normal hearing level
cVEMP: cervical vestibular evoked myogenic potentials
oVEMP: ocular vestibular evoked myogenic potentials
MTDPS7: mitochondrial DNA depletion syndrome 7
IOSCA: infantile-onset spinocerebellar ataxia
PEOA3: autosomal dominant progressive external ophthalmoplegia
