# Supplementary figures and images for: Novel neuro-audiological findings and further evidence for TWNK involvement in Perrault syndrome
Source: J Transl Med. 2017 Feb 8;15:25. doi: 10.1186/s12967-017-1129-4 (PMC5299684; doi:10.1186/s12967-017-1129-4)

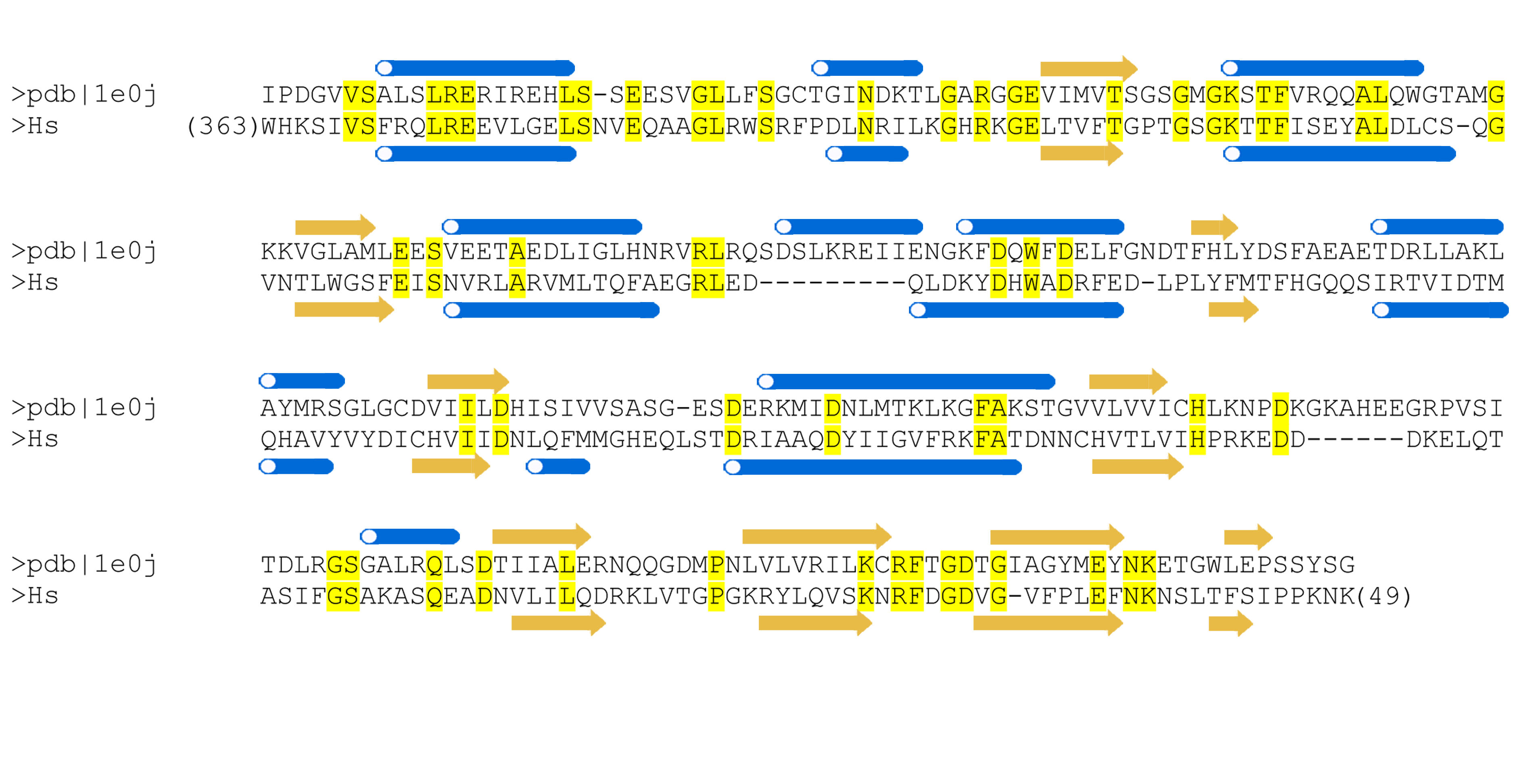

Supplement: Supplementary file 1 — Additional file 1: Figure S1. Sequence-to-structure alignment between the human Twinkle protein (Hs) and the template (PDB code 1e0j). The numbers of residues that are not shown are specified in parentheses. Identical residues are highlighted in yellow. Locations of observed (for 1e0j) and predicted (for Hs) secondary structure elements are marked above and below the corresponding sequences. Helices are presented as blue cylinders, while beta strands as orange arrows. [file 12967_2017_1129_MOESM1_ESM.tif]
